# Insights from the draft genome into the pathogenicity of a clinical isolate of *Elizabethkingia meningoseptica* Em3

**DOI:** 10.1186/s40793-017-0269-8

**Published:** 2017-09-16

**Authors:** Shicheng Chen, Marty Soehnlen, Frances P. Downes, Edward D. Walker

**Affiliations:** 1 Department of Microbiology and Molecular Genetics, Michigan State University, 2215 Biomedical and Physical Sciences Building, 567 Wilson Road, East Lansing, MI 48824-4320 USA; 2 Michigan Department of Health and Human Services, Bureau of Laboratories, Lansing, MI 48906 USA; 3 Biomedical Laboratory Diagnostics Program, Michigan State University, East Lansing, MI 48824 USA

**Keywords:** Draft genome, Infections, *Elizabethkingia meningoseptica*, Human isolate

## Abstract

*Elizabethkingia meningoseptica* is an emerging, healthcare-associated pathogen causing a high mortality rate in immunocompromised patients. We report the draft genome sequence of *E. meningoseptica* Em3, isolated from sputum from a patient with multiple underlying diseases. The genome has a length of 4,037,922 bp, a GC-content 36.4%, and 3673 predicted protein-coding sequences. Average nucleotide identity analysis (>95%) assigned the bacterium to the species *E. meningoseptica.* Genome analysis showed presence of the curli formation and assembly operon and a gene encoding hemagglutinins, indicating ability to form biofilm. In vitro biofilm assays demonstrated that *E. meningoseptica* Em3 formed more biofilm than *E. anophelis* Ag1 and *E. miricola* Emi3, both lacking the curli operon. A gene encoding thiol-activated cholesterol-dependent cytolysin in *E. meningoseptica* Em3 (potentially involved in lysing host immune cells) was also absent in *E. anophelis* Ag1 and *E. miricola* Emi3. Strain Em3 showed α-hemolysin activity on blood agar medium, congruent with presence of hemolysin and cytolysin genes. Furthermore, presence of heme uptake and utilization genes demonstrated adaptations for bloodstream infections. Strain Em3 contained 12 genes conferring resistance to β-lactams, including β-lactamases class A, class B, and metallo-β-lactamases. Results of comparative genomic analysis here provide insights into the evolution of *E. meningoseptica* Em3 as a pathogen.

**Electronic supplementary material:**

The online version of this article (10.1186/s40793-017-0269-8) contains supplementary material, which is available to authorized users.

## Introduction

10.1601/nm.9258, a Gram-negative, aerobic bacillus, belongs to the family 10.1601/nm.8070 within the phylum *Bacteroidaeota* [1–3]. Among the three clinically important 10.1601/nm.9465 species (including 10.1601/nm.9258, 10.1601/nm.22689 and 10.1601/nm.9278), 10.1601/nm.9258 has been intensively investigated for its pathogenicity [4–6]. Most of the 10.1601/nm.9258 infections are nosocomial, often transmitted in intensive care units [1, 7]. This bacterium survives in tap water, in disinfection fluid, on wet surfaces of sinks, in ventilators, hemodialysis equipment, catheters, and other medical apparatus. 10.1601/nm.9258 infection causes neonatal meningitis, nosocomial pneumonia, bacteremia, osteomyelitis, endocarditis, and skin infections [1, 4, 8]. Moreover, older (age > 65) and immunocompromised patients are more susceptible to infection; case-fatality rates have reached 50% [9].

Infections by 10.1601/nm.9258 are difficult to treat with antimicrobial agents due to multiple drug resistance [4]. Tetracycline, chloramphenicol, and β-lactams have been used to treat patients [10], but increasingly clinical isolates lack susceptibility to these antibiotics [11]. Analysis of the resistome in the related bacterium 10.1601/nm.9278 revealed multiple drug resistance genes [12]. Some antibiotics effective against Gram-positive bacteria such as vancomycin, quinolones, tigecycline, and rifampin have been used for treating 10.1601/nm.9258-infected patients, though the mechanism of action remains unclear [12, 13]. Also, the effectiveness of these antibiotics varied; many patients resolved infection but isolates showed high MICs in vitro, thus the relationship between MICs and clinical response was obscure [14]. Further genome analyses will elucidate the breadth of antibiotic susceptibility and resistance mechanisms in 10.1601/nm.9465 spp.

Differentiation of 10.1601/nm.9465 species using routine morphological and biochemical tests is difficult in clinical laboratories [14]. Comparison of 16S rRNA identity does not provide sufficient resolution to identify and separate these closely-related 10.1601/nm.9465 species [2, 14]. Characterization of 10.1601/nm.9465 species by MALDI-TOF mass spectrometry would facilitate it if species reference spectra were added to the database [14]. A limitation is that MALDI-TOF mass spectrometry is not available in many smaller clinical microbiology laboratories. Whole genome analysis facilitates the development of molecular diagnosis tools (such as single nucleotide polymorphisms) that can be potentially useful for small laboratories. In this study, we sequenced, annotated and analyzed a clinical 10.1601/nm.9258 genome, with the aim of providing a better understanding of antibiotic resistance and pathogenesis mechanisms in this pathogen, and of unveiling useful biosystematic molecular markers.

## Organism information

### Classification and features

**Fig. 1:**
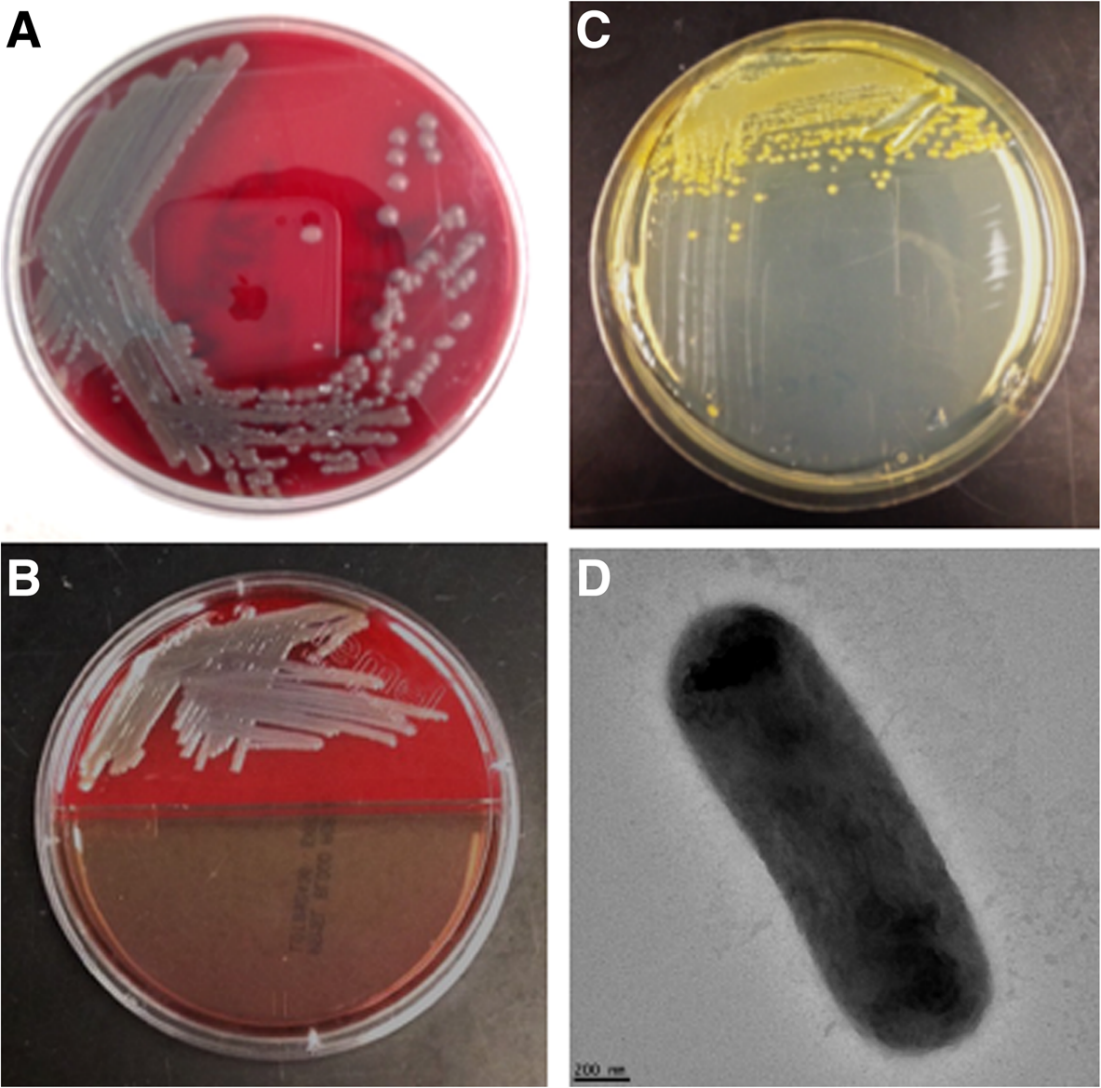
Demonstration of cell growth, pigment production and micrograph. **a** Em3 growing on SBA medium; **b** Demonstration of Em3 grown on SBA medium (control, up part) and MacConkey agar (low part). **c** Pigment production in Em3 grown on TSA agar. **d** Scanning electron microscopy image of Em3

**Fig. 2:**
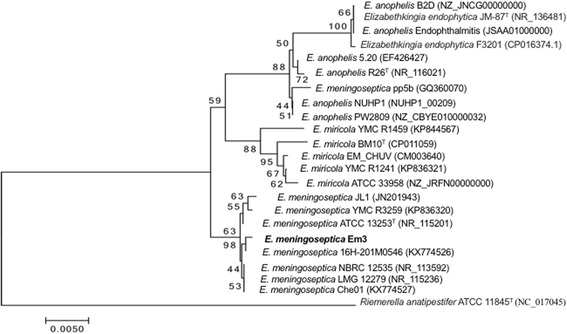
Phylogenetic tree displays the position of *E. meningoseptica* Em3 (shown in bold) relative to the other type strains of *Elizabethkingia* based on 16S rRNA. The phylogenetic tree was constructed by MEGA v. 7.0.14 using the Neighbor-Joining method [42]. The percentage of replicate trees where the associated taxa clustered together in the bootstrap test (500 replicates) is indicated next to the branches. The branch lengths are scaled to the same units as those of the evolutionary distances for inferring the phylogenetic tree. The accession numbers for 16 s rRNA sequences are listed in the parenthesis following selected bacteria: E. meningoseptica LMG 12279 (NR_115236), E. meningoseptica Che01 (KX774527), E. meningoseptica NBRC 12535 (NR_113592), E. meningoseptica ATCC 13253^T^ (NR_115201), E. meningoseptica JL1 (JN201943), E. meningoseptica YMC R3259 (KP836320), E. meningoseptica Em3, E. meningoseptica 16H-201 M0546 (KX774526), E. miricola YMC R1459 (KP844567), E. miricola BM10^T^ (CP011059), E. miricola EM_CHUV (CM003640), E. miricola YMC R1241 (KP836321), E. miricola ATCC 33958 (NZ_JRFN00000000), E. meningoseptica pp5b (GQ360070), E. anophelis NUHP1 (NUHP1_00209), E. anophelis PW2809 (NZ_CBYE010000032), E. anophelis 5.20 (EF426427), E. anophelis R26^T^ (NR_116021), *E. anophelis* Endophthalmitis (JSAA01000000), *Elizabethkingia endophytica* JM-87^T^ (NR_136481), *Elizabethkingia endophytica* F3201 (CP016374.1), E. anophelis B2D (NZ_JNCG00000000) and Riemerella anatipestifer ATCC 11845^T^ (NC_017045)

10.1601/nm.9258 Em3 (Fig. 1) was isolated from a sputum sample from a patient with multiple underlying diseases and on life support. *E. meningoseptica* Em3 is Gram-negative, non-motile and non-spore-forming (Fig. 1 and Table 1). A taxonomic analysis was performed by comparing the 16S rRNA gene sequence to those in the GenBank (Fig. 2). The phylogenetic tree based on the 16S rRNA gene sequences indicated that strain Em3 was clustered within a branch containing other 10.1601/nm.9258 and departing from the clusters 10.1601/nm.22689 and 10.1601/nm.9278 in the genus 10.1601/nm.9465 (Fig. 2). We further calculated the ANI and DDH values among the representative 10.1601/nm.9465 (Table 2). Our results showed that strain Em3 belongs to 10.1601/nm.9258 because of the high ANI (>95%, cutoff for species differentiation) and DDH (>70%, cutoff for species differentiation) values between strain Em3 and 10.1601/nm.9258 10.1601/strainfinder?urlappend=%3Fid%3DATCC+13253 ^T^ [15].

**Table 1:** Classification and general features of E. meningoseptica Em3

MIGS ID	Property	Term	Evidence code^a^
	Classification	Domain *Bacteria*	TAS [37]
		Phylum *Bacteroidaeota*	TAS [3]
		Class *Flavobacteriia*	TAS [38]
		Order *Flavobacteriales*	TAS [39]
		Family *Flavobacteriaceae*	TAS [40]
		Genus *Elizabethkingia*	TAS [2]
		Species *Elizabethkingia meningoseptica*	TAS [2]
		Strain Em3	TAS [2]
	Gram stain	Negative	IDA
	Cell shape	Rod	IDA
	Motility	Non motile	IDA
	Sporulation	Non-spore-forming	NAS
	Temperature range	4–40 °C	IDA
	Optimum temperature	37 °C	IDA
	pH range; Optimum	4–10; 8	IDA
	Carbon source	Heterotroph	IDA
	Energy source	Varied; including glucose and mannitol	IDA
MIGS-6	Habitat	Human	NAS
MIGS-6.3	Salinity	Not determined	
MIGS-22	Oxygen requirement	Aerobic	NAS
MIGS-15	Biotic relationship	Free-living	NAS
MIGS-14	Pathogenicity	Pathogen	NAS
MIGS-4	Geographic location	Michigan, USA	NAS
MIGS-5	Sample collection time	February, 6, 2016	NAS
MIGS-4.1	Latitude	42° 43′ 57″ N	NAS
MIGS-4.2	Longitude	84° 33′ 20″ W	NAS
MIGS-4.4	Altitude	Not reported	NAS

^a^Evidence codes - IDA: Inferred from Direct Assay; TAS: Traceable Author Statement (i.e., a direct report exists in the literature); NAS: Non-traceable Author Statement (i.e., not directly observed for the living, isolated sample, but based on a generally accepted property for the species, or anecdotal evidence). These evidence codes are from the Gene Ontology project [41]

**Table 2:** Percentage of in silico DNA-DNA hybridization (DDH)^a^ and average nucleotide identities (ANI)^b^ among the selected *Elizabethkingia* genomes

	*E. meningoseptica* EM3	*E. anophelis* R26^T^ [43]	*E. meningoseptica* ATCC 13253^T^ [44]	*E. miricola* BM10^T^ [45]	*E. endophytica* JM-87^T^ [46]
*E. meningoseptica* EM3		**31.90** 80.15	**91.10** 98.52	**31.20** 80.44	**32.70** 80.25
*E. anophelis* R26^T^	**31.90** 80.15		**33.60** 80.26	**68.80** 91.52	**78.60** 97.49
*E. meningoseptica* ATCC 13253^T^	**91.10** 98.52	**33.60** 80.26		**31.40** 80.26	**33.30** 80.41
*E. miricola* BM10^T^	**31.20** 80.44	**68.80** 91.52	**31.40** 80.26		**68.70** 91.41
*E. endophytica* JM-87^T^	**32.70** 80.25	**78.60** 97.49	**33.30** 80.41	**68.70** 91.41	

Nucleotide sequences were downloaded from GenBank. The accession numbers for *E. anophelis* R26^T^, *E. meningoseptica* ATCC 13253^T^, *E. miricola* BM10^T^ and *E. endophytica* JM-87^T^ are NZ_ANIW01000001.1, NZ_ASAN01000001.1, NZ_CP011059.1 and NZ_CP016372, respectively

^a^In silico DNA-DNA hybridization was calculated by using Genome-to-Genome Distance Calculator (GGDC) [47]. The percentage of DDH was shown on the top and bolded

^b^ANI values were computed for pairwise genome comparison with using the OrthoANIu algorithm [48]. The percentage of ANI was shown on the bottom

The motility was tested on semi-TSA. The cells of strain Em3 are straight and rods and have a diameter of 0.7 μm and length of 24.0 μm. Strain Em3 grew on TSA, producing yellow pigment (Fig. 1). This bacterium also grew well on SBA with greyish discoloration around the colonies, showing it had the α-hemolytic activity (Fig. 1). 10.1601/nm.9258 Em3 did not grow on MacConkey agar, a finding consistent with strain-dependent growth on this medium; e.g., 10.1601/nm.9258 10.1601/strainfinder?urlappend=%3Fid%3DCCUG+214 ^T^ grew on MacConkey agar whereas other hospital-associated 10.1601/nm.9258 strains did not [2]. Of those strains growing on MacConkey agar, lactose was not utilized [2]. The optimal growth temperature for strain Em3 was 37 °C (Table 1). Carbon source, nitrogen source utilization and osmotic tolerance were assayed by incubating cells in Biolog GEN III microplates at 37 °C overnight (CA, USA). The results showed that 10.1601/nm.9258 Em3 did not tolerate 4% NaCl. 10.1601/nm.9258 Em3 utilized several carbon sources, including D-maltose, D-trehalose, D-gentibiose, D-melibiose, D-glucose, D-mammose, D-fructose, D-fucose, D-mannitol and D-glycerol. The ability to use D-melibiose can differentiate 10.1601/nm.9258 from 10.1601/nm.22689 and 10.1601/nm.9278 [16]. The inability to grow on cellobiose or citrate was consistent with previous reports [16]. Moreover, 10.1601/nm.9258 Em3 utilized D-serine, L-alanine, L-aspartic acid, L-glutamic acid, L-histidine and L-serine when tested on Biolog GEN III microplates.

#### Extended feature descriptions

Phylogenetic analysis (Additional file 1: Figure S1) was further conducted by using 19 genomes with 1181 core genes per genome (22,439 in total). As expected, 10.1601/nm.9258 Em3 grouped together with the selected 10.1601/nm.9258 species and separated from the clusters 10.1601/nm.22689, 10.1601/nm.26899 and 10.1601/nm.9278, a finding similar to the phylogenetic analysis based on 16 s rRNA sequences. Further, both trees (Fig. 1 and Additional file 1: Figure S1) show that species 10.1601/nm.22689 and 10.1601/nm.26899 are not separated well, which is consistent with previous reports [17].

## Genome sequencing information

### Genome project history

The genome of 10.1601/nm.9258 Em3 was selected for whole genome sequencing because of its association with pulmonary disease. Comparison of strain Em3 genome with other 10.1601/nm.9465 species may provide insights into the molecular basis of pathogenicity and metabolic features of this strain. The high-quality draft genome sequence was completed on August 1, 2016 and was deposited to GenBank as a Whole Genome Shotgun project under accession number MDTY00000000 and the Genome OnLine Database with ID Gp0172366 (Table 3).

**Table 3:** Project information

MIGS ID	Property	Term
MIGS 31	Finishing quality	High-quality draft
MIGS-28	Libraries used	two paired-end 250 bp library
MIGS 29	Sequencing platforms	MiSeq-Illumina
MIGS 31.2	Fold coverage	50.0X
MIGS 30	Assemblers	SPAdes 3.9.0
MIGS 32	Gene calling method	NCBI Prokaryotic Genome, Annotation Pipeline
	Locus Tag	BFF93_
	Genbank ID	MDTY00000000.1
	GenBank Date of Release	October 25, 2016
	GOLD ID	Gp0172366
	BIOPROJECT	PRJNA338129
MIGS 13	Source Material Identifier	CL16–200185
	Project relevance	Clinical pathogen

### Growth conditions and genomic DNA preparation

For genomic DNA isolation, *E. meningoseptica* Em3 (CL16–200185, Bureau of Laboratories, Michigan Department of Health and Human Services) culture was grown overnight in 25 mL LB medium at 37 °C with vigorous shaking. DNA was isolated using a Wizard Genomic DNA Purification Kit (Promega, Madison). The amount of genomic DNA was measured using a Nanodrop2000 UV-Vis Spectrophotometer (Thermo scientific) and Qubit DNA assay kit. DNA integrity was confirmed by agarose gel assay (1.5%, *w*/*v*).

### Genome sequencing and assembly

NGS libraries were prepared using the Illumina TruSeq Nano DNA Library Preparation Kit. Completed libraries were evaluated using a combination of Qubit dsDNA HS, Caliper LabChipGX HS DNA and Kapa Illumina Library Quantification qPCR assays. Libraries were combined in a single pool for multiplex sequencing and the pool was loaded on one standard MiSeq flow cell (v2) and sequencing performed in a 2x250bp, paired end format using a v2, 500 cycle reagent cartridge. Base calling was done by Illumina Real Time Analysis [18] v1.18.54 and output of RTA was demultiplexed and converted to FastQ format with Illumina Bcl2fastq v1.8.4.

The Illumina data were assembled into contiguous sequences using SPAdes version 3.9.0 [19], then short contigs (<400 bp) were filtered out. The 11 contigs identified in this strain were therefore submitted to the NCBI database as a Whole Genome Shotgun project.

### Genome annotation

Annotation of the 11 contigs was first done through the NCBI Prokaryotic Genome Automatic Annotation Pipeline [20]. The predicted CDSs were next translated and analyzed against the NCBI non-redundant database, iPfam, TIGRfam, InterPro, KEGG and COG. The RAST system was used to check the annotated sequences [21, 22]. Additional gene prediction and manual revision was performed by using the IMG/MER platform. 10.1601/nm.9258 Em3 genome is available in IMG (genome ID = 2,703,719,242).

## Genome properties

The draft genome sequence is 4,037,922 bp long, 36.37% G + C rich and contains 11 scaffolds (Table 4). Of 3729 genes predicted, 3673 encoded proteins and 56 were RNAs. 2585 (69.32%) were assigned a putative function, while the other 1088 (30.68%) were designated as hypothetical proteins. The distribution of coding genes into general COG functional categories analyzed by IMG is listed in Table 5. Collectively, the genome features were similar to those in other sequenced 10.1601/nm.9258 (Additional file 2: Table S1).

**Table 4:** Genome statistics of *E. meningoseptica* Em3

Attribute	Value	% of total
Genome size (bp)	4,037,922	100
DNA coding (bp)	3,571,073	88.44
DNA G + C (bp)	1,468,714	36.37
DNA scaffolds	11	NA
Total genes	3729	100
Protein coding genes	3673	98.50
RNA genes	56	1.50
Pseudo genes	0	0
Genes in internal clusters	752	20.17
Genes with function prediction	2585	69.32
Genes assigned to COGs	1993	53.45
Genes with Pfam domains	2740	73.48
Genes with signal peptides	452	12.12
Genes with transmembrane helices	818	21.94
CRISPR repeats	0	0

**Table 5:** Number of genes associated with general COG functional categories

Code	Value	%age	Description
J	186	8.58	Translation, ribosomal structure and biogenesis
A	0	0	RNA processing and modification
K	170	7.84	Transcription
L	91	4.20	Replication, recombination and repair
B	0	0	Chromatin structure and dynamics
D	21	0.97	Cell cycle control, Cell division, chromosome partitioning
V	81	3.74	Defense mechanisms
T	82	3.78	Signal transduction mechanisms
M	184	8.49	Cell wall/membrane biogenesis
N	10	0.46	Cell motility
U	17	0.78	Intracellular trafficking and secretion
O	110	5.08	Posttranslational modification, protein turnover, chaperones
C	106	4.89	Energy production and conversion
G	120	5.54	Carbohydrate transport and metabolism
E	184	8.49	Amino acid transport and metabolism
F	60	2.77	Nucleotide transport and metabolism
H	134	6.18	Coenzyme transport and metabolism
I	96	4.43	Lipid transport and metabolism
P	153	7.06	Inorganic ion transport and metabolism
Q	39	1.80	Secondary metabolites biosynthesis, transport and catabolism
R	203	9.37	General function prediction only
S	105	4.85	Function unknown
–	1736	46.55	Not in COGs

## Insights from the genome sequence

**Fig. 3:**
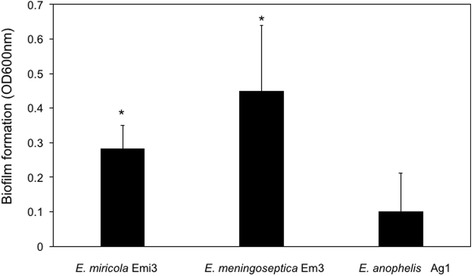
In vitro biofilm assay in the selected *Elizabethkingia* sp. The cells were first cultured by shaking in TSB at 37 °C overnight. The cell density was adjusted to the same OD at 600 nm (0.1). 200 μl of cells were placed on 96-well plates for 24 h. The biofilm assay was carried out using crystal blue staining [49]. Values are mean values for single measurements from eight independent cultures. The error bars are standard deviations. The statistical test was the Student’s t-test. The asterisk indicates a significant difference compared to biofilm formation in E. anophelis Ag1 (*p* < 0.05).

10.1601/nm.9465 bacteria cause sepsis, bacteremia, meningitis or respiratory tract infections in hospitalized patients, indicating that they have the ability to colonize host tissues, suppress the host immune response, and disrupt erythrocytes to obtain nutrients and propagate in the host bloodstream [1, 13, 14]. Genome analysis showed that 10.1601/nm.9258 Em3 carried a gene (BFF93_RS1398) encoding a hemagglutinin protein. Hemagglutinins as adhesins are required for virulence in bacterial pathogens [23]. Hemagglutins facilitate infection via adherence to epithelial cell lines from the human respiratory tract in 10.1601/nm.1747 [24]. Darvish et al. showed that filamentous hemagglutinin adhesins were crucial for bacterial attachment and subsequent cell accumulation on target substrates [25]. An in vitro biofilm assay showed that, compared to the mosquito isolate 10.1601/nm.22689 Ag1, clinical isolates 10.1601/nm.9258 Em3 and 10.1601/nm.9278 Emi3 formed a higher amount of biofilm (Fig. 3). Furthermore, 10.1601/nm.9258 Em3 had better ability to form biofilm than did 10.1601/nm.9278 Emi3. The capacity for strain Em3 to form biofilm was further exemplified by discovery of an operon involved in curli biosynthesis and assembly (BFF93_RS03755, BFF93_RS03760, BFF93_RS03765, BFF93_03725 and BFF93_RS03775). In vitro studies demonstrated that curli fibers participated in bacterial adhesion to target cell surfaces, cell aggregation, as well as biofilm formation [26, 27]. Moreover, some studies showed that curli mediated host cell attachment and invasion in vivo [28]. Curli were involved in inducing the host inflammatory response [29]. It should be noted that this curli synthesis operon is present in 10.1601/nm.9258 while it is absent in 10.1601/nm.9278. Further experiments are warranted to test if the curli gene cluster contributed to biofilm formation in strain Em3 because biofilm formation may involve other genes.

A cytolysin encoding gene (BFF93_RS16990) was found in the strain Em3 genome, whose product belonged to a thiol-activated, CDC family [30]. CDC as a virulence factor is widely distributed among Gram-positive, opportunistic pathogens [31]. For example, 10.1601/nm.5606 utilized pore-forming CDC to translocate a protein into eukaryotic cells [32]. Disruption of expression of a hemolysin (CDC) gene in the intracellular pathogen 10.1601/nm.5096 reduced virulence in mice, showing that CDC was critical for full virulence [33]. Furthermore, perfringolysin, a CDC toxin, has cytotoxicity and leukostasis activities, allowing the cells to escape from macrophage phagosomes during 10.1601/nm.3991 gas gangrene [34]. Only a few CDCs have been found in Gram-negative bacteria [31], and this is the first report of CDC genes in 10.1601/nm.9258. It should also be noted that this cytolysin gene is located immediately downstream of *hmuY*, which together comprise part of an iron metabolism gene cluster. Such gene organization was only seen in 10.1601/nm.9258. This CDC protein sequence in strain Em3 shared 87%, 83% and 81% identity to that in 10.1601/nm.9258 10.1601/strainfinder?urlappend=%3Fid%3DATCC+13253, 10.1601/nm.9258 B2D and 10.1601/nm.9258 10.1601/strainfinder?urlappend=%3Fid%3DNBRC+12535, respectively. It is interesting that it did not have close identity to that in 10.1601/nm.9258 FMS-007 (48%) and 10.1601/nm.9258 502 (48%); it was absent in an 10.1601/nm.9258 strain associated with endophthalmitis [35]. Similarly, it was not conserved in 10.1601/nm.22689 (identity ranging from 0 to 50%) and absent in all 10.1601/nm.9278 species. Such observations may stress that a diverse pathogenesis process exists in various 10.1601/nm.9258 and other 10.1601/nm.9465 species.

Besides a CDC gene in strain Em3 genome, we found that there was a gene encoding the hemolysin with a CBS domain (BFF93_RS14485). Hemolysin can be possibly secreted and involved in lysis of the erythrocytes [35]. The predicted amino acid sequence was conserved in most 10.1601/nm.9258 strains (> 90%). Further examination of hemin-degrading/transporter/utilization proteins led to a discovery of the gene cluster including SAM-dependent methyltransferases (BFF93_RS02055), iron ABC transporter (BFF93_RS02045), hemin-degrading protein (hmuS, BFF93_RS02060), hemin importer ATP-binding protein (BFF93_RS02050) and iron-regulated protein (BFF93_RS02065). Furthermore, there was a gene encoding a hemin receptor (BFF93_RS03140).

*Elizabethkiniga* infections can be fatal in immune-compromised patients if appropriate antibiotic therapy is delayed or the antimicrobial treatment is not properly provided [9, 14]. However, *Elizabethkiniga* spp. are multi-drug resistant [4, 13]. The prediction results by CARD and RAST (Table 6) showed that there are at least 31 genes involved in antibiotic resistance including antibiotic inactivation enzymes and related efflux pumps in 10.1601/nm.9258 Em3. Many of them are possibly involved in mupirocin, vancomycin, β-lactam, aminocoumarin, elfamycin, isoniazid, tetracycline and fluoroquinolone resistance (Table 6). Several drugs used to treat *Elizabethkiniga*-infected patients in the past are not effective anymore [4], which agrees with recent resistome assays in clinical 10.1601/nm.9258 isolates [12]. Genes associated with resistance to β-lactams, aminoglycosides, tetracycline, vancomycin, and chloramphenicol, reported here in strain Em3, are present in most of the studied 10.1601/nm.9465 spp. (Table 6). Remarkably, at least 12 β-lactam resistance genes encoding MBL fold metallo-hydrolases, metallo-β-lactamases and β-lactamases (class A and B) were found in 10.1601/nm.9258 Em3 genome (Table 6). Alternatively, antibiotics such as ciprofloxacin, minocycline, trimethoprim-sulfamethoxazole, rifampin and novobiocin may remain effective due to absence of relevant antibiotic resistance genes in 10.1601/nm.9465 sp. [36]. Therefore, a combination of antimicrobial tests and resistome analysis, combined with rapid identification of infections, will contribute to efficient management for 10.1601/nm.9258 infections in the future.

**Table 6:** Antibiotic genes prediction

			*E. meningoseptica*		*E. anophelis*		*E. miricola*	
Locus number	Gene in Em3	Putative function	Em3	502	R26	NUHP1	ATCC 33958	EM_CHUV
		β-lactam						
BFF93_RS01220	*bla*_GOB-13_	Class B carbapenemase Bla_GOB-13_	+	+	+	+	+	+
BFF93_RS04805	–	β-lactamase	+	+	+	+	+	+
BFF93_RS05700	–	β-lactamase (EC 3.5.2.6)	+	+	+	+	+	+
BFF93_RS07625	*bla*_ACME_	β-lactamase (Bla_ACME_) VEB-1-like	+	+	+	+	+	+
BFF93_RS06860	*bla*_B_	BJP β-lactamase	+	+	+	+	+	+
BFF93_RS09265	–	MBL fold metallo-hydrolase	+	+	+	+	+	+
BFF93_RS06995	–	β-lactamase (EC 3.5.2.6)	+	+	+	+	+	+
BFF93_RS14540	–	β-lactamase	+	+	+	+	–	+
BFF93_RS12085	–	β-lactamase (EC 3.5.2.6)	+	+	+	+	+	+
BFF93_RS12510	–	MBL fold metallo-hydrolase	+	+	+	+	+	+
BFF93_RS14000	*bla*B-9	Class B carbapenemase BlaB-9	+	+	+	+	+	+
BFF93_RS01365	–	β-lactamase (EC 3.5.2.6)	+	+	+	+	+	+
		Sulfonamide						
BFF93_RS00125	dhfR	Dihydrofolate reductase DHFR	+	+	+	+	+	+
BFF93_RS17395	–	Bifunctional deaminase-reductase	+	+	+	+	+	+
BFF93_RS00125	*dhfR*	Dihydrofolate reductase DHFR	+	+	+	+	+	+
BFF93_RS17395	–	Bifunctional deaminase-reductase protein	+	+	+	+	+	+
BFF93_RS14765	*folP*	Dihydropteroate synthase FolP (EC 2.5.1.15)	+	+	+	+	+	+
		Tetracycline						
BFF93_RS08380	*tetA*	Tetracycline efflux protein TetA	+	+	+	+	+	+
BFF93_RS07335	–	Transmembrane efflux protein	+	+	+	+	+	+
BFF93_RS12745	–	Antibiotic transporter	+	+	+	+	+	+
		Macrolide						
BFF93_RS00370	*lolD*	Macrolide resistance, ABC transporter	+	+	+	+	+	+
BFF93_RS05670	*emrB*	Erythromycin resistance, EmrB/QacA	+	+	–	–	+	+
BFF93_RS05670	*emrB*	Erythromycin resistance, EmrB/QacA	+	+	+	+	+	+
BFF93_RS10830	*emrB*	Erythromycin resistance, EmrB/QacA	+	+	+	+	+	+
BFF93_RS03320	–	Erythromycin esterase	+	+	+	+	+	+
		Quinolone						
BFF93_RS04670	*gyrA*	DNA gyrase GyrA subunit A (T83S)	+	+	+	+	+	+
BFF93_RS09245	*gyrB*	DNA gyrase GyrB subunit A (M437 L)	+	+	+	+	+	+
BFF93_RS08895	*parE*	DNA topoisomerase IV subunit B (M437F/A473L)	+	+	+	+	+	+
		Aminoglycoside						
BFF93_RS10790	*ant-6*	Aminoglycoside 6-adenylyltransferase	+	+	+	+	+	+
		Chloramphenicol						
BFF93_RS14765	*catB*	Chloramphenicol acetyltransferase CatB	+	+	+	+	+	+
BFF93_RS04080	*bcr/cflA*	Bcr/CflA efflux pump	+	+	+	+	+	+

“+” or “-” indicates the presence or absence of genes in the selected *Elizabethkingia*

## Conclusions

The draft genome sequence of 10.1601/nm.9258 Em3 isolated from a sputum sample in a patient was sequenced, annotated and described. We found that 10.1601/nm.9258 Em3 had novel genes encoding thiol-activated cholesterol-dependent cytolysin, curli and heme metabolism related proteins, showing that 10.1601/nm.9258 Em3 may be a causative agent. Our results also indicated that 10.1601/nm.9258 might be resistant to β-lactam antibiotics due to the production of diverse MBLs and β-lactamases. Furthermore, these β-lactamase encoding genes were also found in other 10.1601/nm.9465 species, indicating that 10.1601/nm.9465 species were important reservoirs of novel β-lactamase genes. Comparative genomics is a crucial approach in the discovery of novel virulence determinants in 10.1601/nm.9465 species. Genome-based approaches contribute to develop novel genetic markers for future molecular diagnosis of 10.1601/nm.9465 infections.

## Additional files

Additional file 1: Figure S1. Phylogenetic tree of the *Elizabethkingia* genus. The core genome computed by EDGAR 2.0 [50] was extracted to infer a phylogeny for the 18 *Elizabethkingia* genomes. The amino acid sequences of the core genome were aligned using MUSCLE v3.8.31 [51], and then used to construct a phylogenetic tree using the neighbor-joining method as implemented in the PHYLIP package [52]. The accession numbers for genome sequences are listed in the parenthesis following selected bacteria: *E. anophelis* R26^T^ (NZ_ANIW01000066), *E. anophelis* Ag1 (AHHG00000000), *E. endophytica* CSID 3000516978 (NZ_MAHJ01000016), *E. endophytica* F3201 (NZ_MAHU01000055), *E. endophytica* JM-87^T^ (NZ_CP016372), *E. anophelis* NUHP1 (NZ_CP007547), *E. anophelis* CSID 3015183678 (NZ_CP014805), *E. anophelis* FMS007 (NZ_CP006576), *E. miricola* BM10^T^ (NZ_CP011059), *E. miricola* CSID_3000517120 (NZ_MAGX01000009), *E. miricola* GTC862 (NZ_LSGQ01000033), *E. meningoseptica* ATCC 13253^T^ (NZ_ASAN01000115), *E. meningoseptica* G4076 (NZ_CP016376), *E. meningoseptica* CSID_3000515919 (NZ_MAGZ01000024), *E. meningoseptica* EM1 (NZ_MCJH01000010), *E. meningoseptica* EM3 (NZ_MDTY01000011), E. meningoseptica EM2 (NZ_MDTZ01000014), *E. meningoseptica* G4120 (NZ_CP016378), Riemerella anatipestifer ATCC 11845^T^ (NC_017045). (PDF 172 kb)

Additional file 2: Table S1. Genome features for the selected *Elizabethkingia*. Seven genomes from *E. meningoseptica* were provided for comparison of size, gene count, CRISPR, GC and coding count. (XLS 21 kb)

## Abbreviations

ANI: Average nucleotide identities
CDC: Cholesterol-dependent cytolysin
IMG/MER: Integrated Microbial Genomes and Microbiome Samples Expert Review
MALDI-TOF: Matrix assisted laser desorption-ionisation time-of-flight
MBL: Metallo-β-lactamase
MIC: Minimum inhibitory concentration
SBA: Sheep blood agar
TSA: Solid trypticase soy agar
