# Supplementary figures and images for: Insights from the draft genome into the pathogenicity of a clinical isolate of Elizabethkingia meningoseptica Em3
Source: Stand Genomic Sci. 2017 Sep 16;12:56. doi: 10.1186/s40793-017-0269-8 (PMC5602931; doi:10.1186/s40793-017-0269-8)

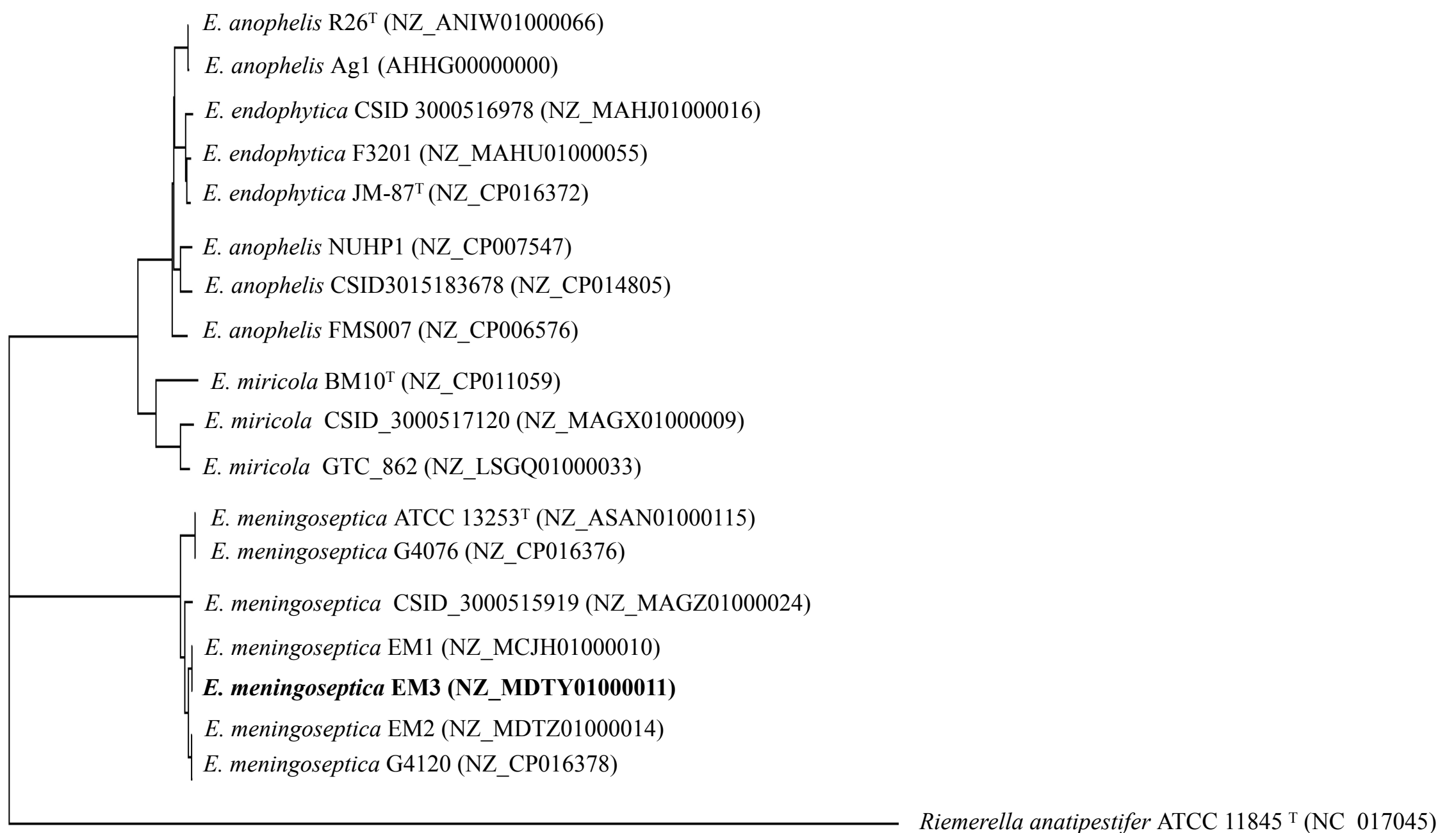

0.01

Supplement: Supplementary file 1 — Phylogenetic tree of the Elizabethkingia genus. The core genome computed by EDGAR 2.0 [50] was extracted to infer a phylogeny for the 18 Elizabethkingia genomes. The amino acid sequences of the core genome were aligned using MUSCLE v3.8.31 [51], and then used to construct a phylogenetic tree using the neighbor-joining method as implemented in the PHYLIP package [52]. The accession numbers for genome sequences are listed in the parenthesis following selected bacteria: E. anophelis R26T (NZ_ANIW01000066), E. anophelis Ag1 (AHHG00000000), E. endophytica CSID 3000516978 (NZ_MAHJ01000016), E. endophytica F3201 (NZ_MAHU01000055), E. endophytica JM-87T (NZ_CP016372), E. anophelis NUHP1 (NZ_CP007547), E. anophelis CSID 3015183678 (NZ_CP014805), E. anophelis FMS007 (NZ_CP006576), E. miricola BM10T (NZ_CP011059), E. miricola CSID_3000517120 (NZ_MAGX01000009), E. miricola GTC862 (NZ_LSGQ01000033), E. meningoseptica ATCC 13253T (NZ_ASAN01000115), E. meningoseptica G4076 (NZ_CP016376), E. meningoseptica CSID_3000515919 (NZ_MAGZ01000024), E. meningoseptica EM1 (NZ_MCJH01000010), E. meningoseptica EM3 (NZ_MDTY01000011), E. meningoseptica EM2 (NZ_MDTZ01000014), E. meningoseptica G4120 (NZ_CP016378), Riemerella anatipestifer ATCC 11845T (NC_017045). (PDF 172 kb) [file 40793_2017_269_MOESM1_ESM.pdf]
